# Is There a Correlation between New Scoring Systems and Systemic Inflammation in Stable Bronchiectasis?

**DOI:** 10.1155/2017/9874068

**Published:** 2017-11-15

**Authors:** Hikmet Coban, Adil Can Gungen

**Affiliations:** ^1^Department of Chest Disease, Sakarya Training and Research Hospital Sakarya, Adapazan, Turkey; ^2^Department of Chest Disease, Istinye University Istanbul, Istanbul, Turkey

## Abstract

**Aim:**

The present study aimed to investigate the relation between FACED and BSI scores, which were developed to measure the severity of bronchiectasis, and systemic inflammation in patients with stable bronchiectasis.

**Methods:**

FACED and BSI scores of 117 patients with stable bronchiectasis were calculated. The correlations between mean scores and CRP levels, leukocyte count, and neutrophil/lymphocyte ratio were investigated.

**Findings:**

Mean BSI and FACED scores were 7.2 ± 5.2 and 2.1 ± 1.8, respectively. The severity of bronchiectasis as determined based on BSI and FACED increased significantly with increasing levels of CRP in patients with stable bronchiectasis (*p*=0.001 and *p*=0.027, resp.). No significant changes were found in leukocyte count (*p*=0.72 and *p*=0.09, resp.) and N/L ratio (*p*=0.45 and *p*=0.71, resp.). BSI and FACED scores were significantly correlated with CRP but not with leukocyte count or N/L ratio.

**Conclusion:**

In patients with stable bronchiectasis who are evaluated based on FACED and BSI scores, CRP can be a useful biomarker as a direct indicator of the severity of systemic inflammation.

## 1. Introduction

Bronchiectasis is a chronic airway disease associated with symptoms such as cough, sputum production, and hemoptysis, and it may develop as a result of various etiologies [1]. A vicious cycle, based predominantly on airway infections, airway inflammation, and structural damage, plays a role in progression and pathogenesis of bronchiectasis [2]. It is disappointing that there are a limited number of effective treatment strategies and evidence-based management recommendations for assessment and follow-up of bronchiectasis. The severity of bronchiectasis should be determined to obtain better treatment outcomes. Assessment of bronchiectasis may be a challenge since there is actually no simple measurement method that has been sufficiently validated. Conventionally, previous reports measured the severity of disease based on FEV1 (forced expiratory volume in 1 s) [3], while Reiff and Bhalla scores in HRCT were also used in some studies to measure disease severity [4, 5]. However, FEV1 was not effective in terms of clinical decision-making, and HRCT (high-resolution computed tomography) scores had a poor correlation with lung functions [6]. Therefore, it became an obligation to create a new scoring system to assess the severity of bronchiectasis. FACED score (F: FEV1, A: age, C: colonization, E: number of affected lobes, and D: dyspnea) [7] and Bronchiectasis Severity Index (BSI) [8] were recently designed as two multidimensional severity grading scales to assess the prognosis of bronchiectasis. FACED score is a five-item grading system that predicts mortality in patients who had been monitored for 5 years. BSI is a seven-item scale that describes future risk of death, hospitalization, and exacerbations. Elevation of systemic inflammatory markers such as C-reactive protein (CRP) and total white blood cell count is known to be associated with the extent of the disease and poor lung functions [9]. The relation between CRP or total white blood cell count and the novel scoring systems used to assess bronchiectasis severity, namely, BSI and FACED, has not been investigated before.

Bronchiectasis is characterized by airway inflammation accompanied by continuous predominance of neutrophils. Proinflammatory cytokines such as IL-1, IL-6, and TNF-a increase, while anti-inflammatory cytokines such as IL-10 decrease in patients with bronchiectasis [10]. As a result of a series of complex interactions between various cells including the neutrophils and lymphocytes, inflammation may eventually cause permanent tissue damage [11]. The ratio of inflammatory cells in blood has a potential to reflect chronic inflammation and pathologic conditions, and the neutrophil/lymphocyte ratio was recently shown to be increased during COPD exacerbations compared to stable disease conditions [12]. In the presence of various chronic conditions such as disorders of the cardiovascular and renal systems, it was shown that increased N/L ratio can be used as a marker of inflammation, and a higher N/L ratio was associated with disease severity, mortality, and hospitalization [13–15]. Since the lungs of patients with bronchiectasis are characterized by neutrophilic inflammation, N/L ratio can also be a useful marker to assess the clinical status of patients with bronchiectasis. The neutrophil/lymphocyte ratio is an inflammation marker that has not been previously investigated in patients with stable bronchiectasis. N/L ratio is an easily accessible marker that can be calculated from the total blood count, and it does not require any special equipment or specific method of measurement. N/L ratio can become an easy-to-use method and a useful marker in patients with bronchiectasis if it proves to be useful as a biological marker in this patient group.

The present study aimed to investigate the relation of CRP, total white blood cell count, and neutrophil/lymphocyte ratio with BSI and FACED scores.

## 2. Methods

Patients who had been followed up by Pulmonology Clinics of Sakarya Training and Research Hospital and had a definitive diagnosis of bronchiectasis based on HRCT and BTS guidelines were included to the study [16]. *The diagnosis of bronchiectasis with HRCT is based on radiology reports. BTS guideline mentions investigation and management of patients with bronchiectasis. BTS guideline does not include bronchiectasis caused by cystic fibrosis* [16]. Patients with immunodeficiency, allergic pulmonary aspergillosis, bronchiectasis secondary to primary ciliary dyskinesia, heart failure, malignancy or chronic renal failure; patients who had used antibiotics or steroids within the last one month; patients who described an acute exacerbation; and pregnant patients were excluded from the study. A total number of 117 patients who met the above-described criteria were included to the study. These patients were followed between 2014 and 2016. The patients who had a history of steroid and antibiotic use in the last month were evaluated in the stabilization period of bronchiectasis. All patients performed spirometry, and peripheral venous blood samples were obtained from all patients on the day of spirometry. Total blood count and CRP levels were analyzed. In patients who were following up for two years, at least two measurements of CRP, WBC, and N/L were taken in the stabilization period, and the averages of these measurements were calculated. For the measurement of serum CRP levels, 3 cc of venous blood samples were collected in biochemistry test tubes. Blood samples were transferred to the analyzing laboratory at most 2 hours after sampling, and the analyses were performed on the same day. CRP levels were quantitatively analyzed by the nephelometric method using the BN 200 device. The reference range in healthy humans for the preferred method of analyses was 0.00–5.00 mg/L.

### 2.1. FACED and BSI Scoring and Grading

FACED score consists of 5 variables: FEV 1%, age, *Pseudomonas aeruginosa* colonization, radiological extent of the disease, and dyspnea as assessed by the Medical Research Council (MRC) scale. The total score is calculated by adding up the scores for each variable and varies between 0 and 7 points. Based on the total score, bronchiectasis is evaluated in three groups as mild (0–2), moderate [3, 4], and severe [5–7] bronchiectasis.

BSI score consists of 9 variables: age, body mass index, FEV1%, hospitalizations within the last two years, number of exacerbations within the last one year, dyspnea as assessed by MRC scale, *Pseudomonas aeruginosa* colonization, colonization by other microorganisms, radiological extent of the disease, and/or presence of cystic bronchiectasis. Total score varies between 0 and 26 points. In BSI, scores of 0–4 points indicate mild, 5–8 indicate moderate, and 9 or above indicate severe bronchiectasis.

### 2.2. Statistical Methods

SPSS for Windows 18.0 (Chicago, IL) software package was used for statistical analyses. Continuous variables were presented as mean values and standard deviations. Differences between two groups were tested by *t*-test for normally distributed variables and by Mann–Whitney test for nonnormally distributed variables. For analysis of more than two groups, ANOVA test was used for normally distributed variables and Kruskal–Wallis test was used for nonnormally distributed variables.

Correlations were evaluated by means of Spearman's rank correlation test. A *p* value less than 0.05 was considered as significant.

## 3. Results

Table 1 shows the characteristics of 117 patients with stable bronchiectasis who were included in this study. Mean FACED and BSI scores and numbers of patients with mild, moderate, and severe bronchiectasis based on both scoring systems are shown in Tables 2 and 3.

**Figure 1:**
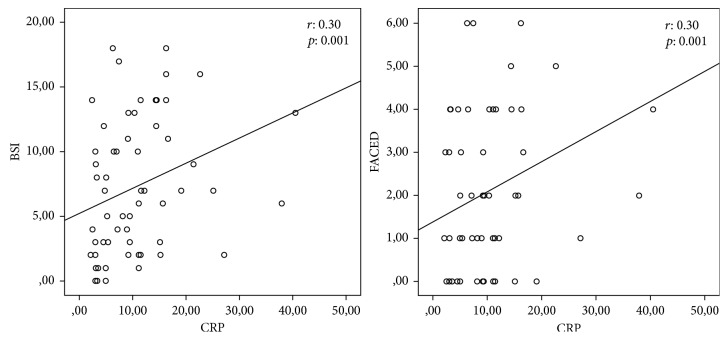
Correlation FACED and BSI scores with CRP levels.

When the patients were classified as those with mild, moderate, and severe bronchiectasis according to BSI and FACED scores, significant relations were found between FVC%, FEV1%, FEV1/FVC, CRP, BMI, number of exacerbations, and number of hospitalizations according to both scoring systems. WBC and N/L ratio were not significantly related to disease severity based on neither of the scoring systems (Tables 2 and 3). A significant positive correlation was found between FACED and BSI scores and CRP levels (Figure 1), and a significant negative correlation was noted between pulmonary function test parameters and BMI index. No significant correlation was demonstrated between FACED and BSI scores and N/L ratio or leukocyte count (Table 4). *Comorbid conditions which may have an effect on systemic inflammation and the CRP levels are shown in*Table 5*. There was no significant difference between the groups.*

## 4. Discussion

In the present study, the severity of bronchiectasis was assessed based on the recently developed FACED and BSI scoring systems, while systemic inflammation was evaluated by CRP levels, leukocyte count, and N/L ratio. Analyses of the correlations between FACED and BSI scores and markers of systemic inflammation in patients with stable bronchiectasis demonstrated a significant relation only with CRP levels, whereas no significant correlation was found with leukocyte count or N/L ratio (Table 4).

A study assessing the severity of bronchiectasis based on HRCT score reported a significant correlation between HRCT score and markers of systemic inflammation such as WBC and CRP [9]. In a study involving patients with diseases that result in respiratory failure, patients with bronchiectasis were shown to have higher CRP levels compared to the other patient groups, and CRP levels were associated with mortality in that study [17]. Patients with bronchiectasis and elevated CRP levels showed rapid FEV1 declines in another study [18]. All these findings suggest that, as long as bronchiectasis is accompanied by systemic inflammation, systemic inflammation should presumably be associated with disease severity. Previous studies assessed the severity of bronchiectasis based on FEV1 or HRCT scores. No previous study demonstrated a correlation between CRP and the recently developed BSI or FACED scoring systems. In the present study, CRP had a significant positive correlation both with BSI (*r* = 0.30, *p*=0.001) and FACED (*r* = 0.30, *p*=0.001) scores. Contrary to our findings, a previous study comparing patients with bronchiectasis to a control group did not identify any finding indicative of systemic inflammation in the bronchiectasis group, and the authors highlighted that increased WBC count and CRP levels in patients with colonization might suggest presence of an inflammatory response [19].

It has been emphasized that increased bronchial inflammation may be present even during periods of clinical stability. During exacerbations, neutrophils migrate to the airways and proteolytic activity increases. Proteolytic agents destroy the lung matrix and contribute to the development of bronchiectasis [20]. In patients with stable bronchiectasis, presence of neutrophilic inflammation in sputum was suggested to be a good biomarker of disease severity. In that study, disease severity was assessed based on lung functions and BSI [21]. Considering there is an inflammation in the lungs with neutrophil predominance, demonstration of this condition in systemic circulation can be a useful parameter to grade the disease severity. N/L ratio was assessed as a marker of systemic inflammation in the presence of chronic diseases involving cardiovascular and renal systems [13–15]. Since bronchiectasis is also a chronic disease, one might expect that the increased neutrophilic inflammation in the lungs might be associated with an elevated N/L ratio in systemic circulation with increasing disease severity. No previous study in the literature investigated N/L ratio in patients with stable bronchiectasis. In the present study, a significant correlation was not found between N/L ratio and FACED or BSI scores in patients with stable bronchiectasis.

In a study performed on patients with last-stage respiratory disorders, including 33 patients with bronchiectasis, a clear relation was demonstrated between decreased BMI and increased mortality [18]. Similarly, a Turkish study suggested that higher BMI values could be beneficial on expected survival time in patients with bronchiectasis [22]. BMI was associated with the findings that reflect disease severity. Patients with low BMI values were shown to more frequently experience acute exacerbations, have poor lung functions, have increased systemic inflammation, and more commonly have chronic *P. aeruginosa* colonization. BMI was emphasized to be one of the factors with the most significant effects on the risk of hospitalization and death [18, 22]. A positive correlation was demonstrated between high BMI and survival [23]. A recent study reported a relation between radiological progression and low BMI [24]. In the present study, significant relations were found between BMI and both BSI and FACED severity classifications reflecting the severity of bronchiectasis. Both scoring systems had significant negative correlations with BMI, while the correlation with FACED (*r* = −0.32, *p*=0.002) score was more evident than the correlation with BSI (*r* = −0.21, *p*=0.047) score.

A recently published study reported that low FVC values represented a risk factor for bacterial colonization [25]. Both BSI and FACED scorings take into account the FEV1 values. Since both scoring systems had significant negative correlations with FVC in the present study (FACED (*r* = −0.70, *p* < 0.0001), BSI (*r* = −0.62, *p* < 0.0001)), it should be considered that the risk of bacterial colonization may increase with numeric increases in both scores, in other words, with increasing disease severity.

Patient distribution was different when the disease severity was classified as mild, moderate, or severe based on the two scoring systems in the present study. On the contrary, Minov et al. [26] reported similar patient distribution rates based on both scoring systems (Tables 2 and 3). To explain this, in a recently published study, Guan et al. emphasized that the difference in FEV1 distribution and the scoring for previous hospitalizations resulted in overall higher BSI scores; therefore, some patients were given a higher severity grade. The authors underlined the need for additional studies comparing effectiveness of grading systems in bronchiectasis [27].

This study has some limitations. Since the study was performed as a single-center study with a small sample size, study results can only be generalized to a limited extent. Moreover, the groups were not homogenous in terms of severity grading based on the two scoring systems.

## 5. Conclusion

In patients with bronchiectasis, CRP can be a useful biomarker that directly reflects the level of systemic inflammation. WBC and N/L ratio failed to capture systemic inflammation in patients with stable bronchiectasis whose severity grading was done based on FACED and BSI scoring systems. Additional studies are required to elucidate the clinical significance of the role of CRP in the assessment of treatment response and progression of bronchiectasis after anti-inflammatory therapy.

## Figures and Tables

**Table 1:** Characteristics of the study participants.

Characteristics	Patients (*n* = 117)
Male/female (*N*%)	65/52 (55.6/44.4)
Mean age (years)	53 ± 15.1
BMI (kg/m^2^)	27.3 ± 5.6
FVC (%)	64.2 ± 21.2
FEV1 (%)	55.5 ± 21.2
FEV1/FVC (%)	70.1 ± 13.7
Pack-years smoked	9.7 ± 14.8
Ex-smokers, *N* (%)	30 (25.6)
Never smokers, *N* (%)	68 (58.1)
Current smokers, *N* (%)	19 (15.3)
CRP (mg/L)	10.4 ± 8.0
N/L ratio	2.2 ± 1.5
WBC (/mm^3^)	8449 ± 2449
*Pseudomonas aeruginosa*, *N* (%)	11 (9.4)
Other microorganisms, *N* (%)	11 (9.4)
Number of affected lobes	2.6 ± 1.0
Exacerbations in previous year	2.0 ± 2.3
Number of hospitalizations in the last two years	0.3 ± 0.6

BMI, body mass index; FVC, forced vital capacity; FEV1, forced expiratory volume in 1 second; FEV1/FVC, forced expiratory volume in 1 second/forced vital capacity; N/L ratio, neutrophil/lymphocyte ratio.

**Table 2:** The relation between bronchiectasis severity based on BSI score and the other study variables.

BSI score = 7.2 ± 5.2				
	Mild (*n* = 46)	Moderate (*n* = 25)	Severe (*n* = 46)	*p*
Age (years)	47.1	56.1	57.2	0.003
FVC (%)	82.6	55.0	52.1	<0.0001
FEV1 (%)	74.7	46.7	42.5	<0.0001
FEV1/FVC (%)	74.4	71.0	65.8	0.020
CRP (mg/L)	6.8	12.9	12.5	0.001
WBC (/mm^3^)	8224	8631	8574	0.727
N/L ratio	2.36	2.40	2.08	0.456
Smoking (pack-years)	9.2	12.0	9.0	0.718
BMI (kg/m^2^)	29.5	25.4	26.2	0.008
Exacerbations in previous year	0.28	2.44	3.65	<0.0001
Number of hospitalizations in the last two years	0.00	0.40	0.95	<0.0001

FVC, forced vital capacity; FEV1, forced expiratory volume in 1 second; FEV1/FVC, forced expiratory volume in 1 second/forced vital capacity; N/L ratio, neutrophil/lymphocyte ratio; BMI, body mass index.

**Table 3:** The relation between bronchiectasis severity based on FACED score and the other study variables.

FACED score = 2.1 ± 1.8				
	Mild (*n* = 71)	Moderate (*n* = 34)	Severe (*n* = 12)	*p*
Age (years)	48.3	56.6	70.1	<0.0001
FVC (%)	76.1	51.4	40.0	<0.0001
FEV1 (%)	68.2	39.9	35.0	<0.0001
FEV1/FVC (%)	74.0	61.0	73	<0.0001
CRP (mg/L)	8.8	12.4	13.8	0.027
WBC (/mm^3^)	8190	9197	7858	0.096
N/L ratio	2.26	2.35	2.01	0.715
Smoking (pack-years)	8.4	12.0	12.0	0.488
BMI (kg/m^2^)	28.6	24.5	27.4	0.007
Exacerbations in previous year	1.3	3.2	3.0	<0.0001
Number of hospitalizations in the last two years	0.15	0.64	1.0	<0.0001

FACED (F: forced expiratory volume in 1 second, A: age, C: colonization, E: number of affected lobes, D: dyspnea); FVC, forced vital capacity; FEV1, forced expiratory volume in 1 second; FEV1/FVC, forced expiratory volume in 1 second/forced vital capacity; N/L ratio, neutrophil/lymphocyte ratio; BMI, body mass index.

**Table 4:** Correlation coefficients and *p* values for FACED and BSI scores and study variables.

	FACED score		BSI score	
	*r*	*p*	*r*	*p*
FVC (%)	−0.70	<0.0001	−0.62	<0.0001
FEV1 (%)	−0.73	<0.0001	−0.67	<0.0001
WBC (/mm^3^)	0.16	0.075	0.01	0.850
CRP	0.30	0.001	0.30	0.001
N/L ratio	−0.02	0.822	−0.11	0.238
BMI (kg/m^2^)	−0.32	0.002	−0.21	0.047

FACED (F: forced expiratory volume in 1 second, A: age, C: colonization, E: number of affected lobes, D: dyspnea); BSI, Bronchiectasis Severity Index; FVC, forced vital capacity; FEV1, forced expiratory volume in 1 second; N/L ratio, neutrophil/lymphocyte ratio.

**Table 5:** Comorbid conditions, which may have an effect on systemic inflammation and the CRP levels.

	CRP (mg/L)	*p*
Total (*n* = 117)	10.4 ± 8.0	0.30
COPD (*n* = 23)	12.8 ± 6.9	
Asthma (*n* = 38)	10.0 ± 7.6	
Hypertension (*n* = 7)	9.8 ± 7.1	
DM (*n* = 6)	4.9 ± 1.4	
None (*n* = 43)	10.2 ± 9.4	

COPD, chronic obstructive pulmonary disease; DM, diabetes mellitus; CRP, C-reactive protein.
